# The Indications for Operation in Myo-Fibroma of the Uterus

**Published:** 1904-09

**Authors:** James Swain

**Affiliations:** Professor of Surgery at University College, Bristol; Surgeon to the Bristol Royal Infirmary


### THE INDICATIONS FOR OPERATION IN MYO-FIBROMA OF THE UTERUS.

BY

JAMES SWAIN, M.S., M.D. Lond., F.R.C.S.,

*Professor of Surgery at University College, Bristol; Surgeon to the Bristol Royal Infirmary.*

WHILST there are some who appear to advise the removal of all myo-fibromatous tumours of the uterus, others incline to non-operative treatment throughout. More lives will, however, be saved by a judicious selection of cases for the respective forms of treatment.

The mere presence of a tumour is not a sufficient excuse for operation, and it is clearly the duty of every surgeon, before he advocates the removal of a myo-fibroma, to show either that it is dangerous to life or that it produces so much discomfort as to render existence more or less intolerable.

My remarks are limited to those cases of myo-fibromata which require treatment, if necessary, by abdominal section.

Matthews Duncan[1] says: "I am sure the fatal number of cases is greater than is generally supposed;" and it will help to elucidate the reasons for surgical interference given below if we briefly consider the chief causes of death occurring in cases that have not been subjected to operation:—

1. *Pressure Effects*.—I have been called upon several times to operate for intestinal obstruction in cases of neglected myo-fibroma, and the fatal results from pressure on the urethra or ureters are self-evident.
2. *Hemorrhage*.—The patient may die as a direct result of excessive bleeding, or indirectly from extreme anæmia.
3. *Peritonitis*.—Necrotic changes may occur in the tumour, leading to septic infection of the peritoneum.
4. The occurrence of *pregnancy* in association with a myo-fibroma is a very serious condition, not only from abortion, which is likely to occur, but also from the interference with natural delivery and hemorrhage.

There are other less common causes of death, but the list is sufficiently long to show that myo-fibromata under certain conditions gravely imperil the life of the patient.

A great deal of misunderstanding exists as regards the influence of the menopause on myo-fibromata; women are so often led to suppose that when once the "change of life" occurs all their troubles will cease, and much valuable time is thus often lost by relying upon a false hope. Not only is the menopause often, indeed generally, delayed for some eight or ten years at least, but in many cases the tumour grows more rapidly at the time when it is usually supposed that a diminution in size may be expected.

Much depends upon whether the myo-fibroma is of the "hard" or "soft" varieties. In the hard myo-fibroma, which is comparatively non-vascular, the rate of growth is slow; the tumour enlarges temporarily with menstruation and pregnancy, and after pregnancy and during the menopause it generally shares in the physiological involution of the uterus which then occurs. Such tumours are most frequently multiple. On the other hand, the soft myo-fibroma, which is usually single, is

[1] *Clinical Lectures on Diseases of Women*, 3rd edition, 1886.

much more vascular; the vessels penetrate the tumour itself, as well as ramifying in the capsule, and give a pinkish colour to this variety of tumour. Associated with this increased vascularity we find a more rapid growth, which appears to be independent of uterine conditions and unaffected by the menopause. It is this form which so commonly becomes larger as the functional activity of the uterus declines.

It is therefore highly necessary that the late occurrence of the menopause and the uncertain effect of that epoch on many of these tumours should be borne in mind when we are dealing with a case of myo-fibroma in a patient who considers that under normal circumstances the final cessation of menstruation should soon occur.

The mere **size of the tumour** may, under certain circumstances, afford a sufficient indication for operative interference. It is commonly ten or twelve years before a myo-fibroma reaches the size of a pregnant uterus at full term, but when the tumour extends to a point two or three inches above the umbilicus it frequently causes considerable annoyance from its bulk even apart from any definite or important pressure symptoms. A comfortable existence is scarcely possible in many of these cases, and commonly, in addition to the inconvenience of getting about and attending to the duties of life, a condition of chronic invalidism is induced by repeated hemorrhages and more or less constant pain. In the more wealthy classes a sufficiently tolerable existence may be dragged out on a sofa, but in others where a necessary wage-earning capacity is seriously interfered with the risk of operation is generally considered preferable. In single women, too, much mental distress may be caused by the comments evoked by the prominence of the abdomen, and the unjust suspicions to which they are subjected may induce them to seek the relief which removal of the tumour affords.

**Hemorrhage** is one of the commonest symptoms of myo-fibromata, and is also one of the most important indications for operation on account of the insidious way in which the patient's health and strength are gradually undermined by its repetition. It is, however, quite remarkable how easily a woman recovers from a very considerable loss of blood, though occurring again and again, and therefore we must not be led to advise operation simply because there is a severe menorrhagia.

Profuse menstrual hemorrhage occurs in about 50 per cent. of cases of myo-fibromata. It is associated with all forms of these tumours—the large and small, hard and soft, sub-peritoneal, interstitial and sub-mucous—though doubtless it is more common and more severe as the tumour approaches the uterine mucosa.

The size of the tumour is not necessarily indicative of the amount of blood lost. At first the "periods" occur at regular intervals, but the flow is excessive, and later on with the growth of the tumour the flow is of longer duration, the amount lost is increased, and the intervals between the periods are shortened. Definite "floodings" may also occur. In the early days the patient fully recovers in the intervals the effects of the excessive flow at the periods, but with the progress of the disease she becomes increasingly anæmic and utterly prostrate.

Recognising the very severe effects of prolonged hemorrhage, we have to decide when operation is required in these cases.

In minor degrees of hemorrhage ergot and chloride of calcium are of service, and packing the vagina will generally effectually stop the flow and so conserve the patient's strength.

The blood comes from the increased surface of the endometrium (for the uterine cavity is enlarged), but hemorrhage is also favoured by the endometritis which so commonly accompanies the growth of the tumour. This perhaps explains the highly satisfactory relief which is afforded by curettage, though this operation is impracticable in cases where there is much displacement and the uterine canal tortuous. In the performance of curettage the strictest antiseptic precautions are necessary, or necrotic changes in the tumour may result and lead to a fatal septicæmia.

Some of these less severe measures are more valuable than is generally supposed, but where they have failed and the patient is steadily losing ground there should be no hesitancy in resorting to a radical operation. This should be advised when the patient ceases to regain in the intervals the loss of strength incidental to the excessive flow at the periods. As soon as we are convinced of this fact operation should be undertaken. Delay means further prostration, and jeopardises the successful result of removal of the tumour.

The occurrence of **pain** as an indication for operation presents some difficulty. Most fibroids give rise to a sense of dragging and some tenesmus, and these are increased at the menstrual epoch owing to an exaggeration of the vascular engorgement which normally takes place at that period. But even severe and persistent dysmenorrhœa must be accepted with great caution as a plea for radical cure, for the degree of pain is difficult to gauge and is apt to be unduly emphasised in neurotic subjects.

It is somewhat different, however, with a persistent inter-menstrual pain. This is often associated with pressure symptoms in the pelvis, or with degenerative changes in the tumour which will presently be considered.

A diseased condition of the ovaries and tubes frequently coexists with myo-fibromata. Pelvic peritonitis, resulting in the fixation of ovaries and tubes in the pelvis, with hydrosalpinx or pyosalpinx, may occasion attacks of pain, or the pain may be due to dragging on the adhesions so produced.

At all events, the development of pain in a case which has hitherto caused no trouble, or any marked increase of pain, requires serious consideration, and where pain of a more or less persistent character is so distressing as to produce a condition of chronic invalidism in spite of sedatives, rest and massage, a radical operation is indicated.

A **rapid enlargement** of the tumour, especially if associated with pain, may almost certainly be regarded as demanding operative treatment. Such symptoms are generally indicative of serious degenerative changes in the tumour itself. Of these there are many, but they can only be briefly referred to here. There are true cysts formed by glandular elements within the tumour, and lined by columnar epithelium; cysts produced by dilatation of the systemic or lymphatic vessels, and lined by endothelium; pseudo-cysts resulting from hemorrhages, suppuration, myxomatous and other changes, and not lined by epithelium; true degenerations, such as fatty, calcareous, and myxomatous degenerations, suppuration and gangrene; and transition into other forms of growth, as sarcoma and carcinoma. I have certainly seen myo-fibromata associated with malignant disease some four or five times in as many years, so it cannot be regarded as of so infrequent occurrence as was at one time supposed. Elevation of temperature and other constitutional disturbance sometimes occur with the rapid increase in size of the tumour, and in cases associated with suppuration or sloughing the patient may exhibit all the phenomena of a severe sapræmia.

The enumeration of these various forms of degeneration will serve to emphasise the importance of rapid growth of the tumour as an indication for prompt radical treatment.

Practically it may be said that operative interference is required if examination at intervals of three months shows a myo-fibroma to be rapidly increasing in size, and that it has reached nearly up to the level of the umbilicus.

The discomfort and chronic invalidism produced by large myo-fibromata are partly dependent upon the **pressure effects** due to the presence of a tumour occupying so much space. As a consequence the intestines, stomach, liver, and diaphragm are pushed upwards, and considerable interference with circulation and digestion occurs. Flatulence is often very distressing. In other cases pressure upon the ureters results in hydronephrosis or other changes in the kidney, and pressure on the bladder causes frequent micturition or perhaps cystitis. The necessity for removal in these large tumours has already been considered.

Even worse pressure effects are produced by smaller tumours incarcerated in the pelvis and prevented from expansion upwards by the sacral prominence. Pressure on the urethra may cause complete retention, and the use of the catheter may be necessary. Severe pain arises from pressure on nerves causing a false sciatica, and in all cases of obscure pain in the region supplied by the sacral nerves the pelvis should always be carefully examined. Pressure on the ovaries, especially if prolapsed, causes a pain of a sickening character, and results in those pathological conditions of the ovaries and tubes which so often accompany myo-fibromata and to which reference has been made. Pressure on the rectum causes piles and severe constipation, amounting in some cases to a definite obstruction, though it is remarkable how great a degree of pressure the rectum will tolerate without causing urgent symptoms.

In many of these cases the tumour can be displaced above the pelvic brim, with consequent relief of symptoms, much in the same way as a retroverted gravid uterus can be rectified; but great care must be exercised in the manipulation, lest an undue traumatism may cause tearing of adhesions or degenerative changes of a serious nature in the tumour itself. If rectification is impossible or undesirable a radical operation should be undertaken without delay, but it is highly desirable that we should not wait for the development of urgent symptoms when we have ascertained that there is a growing tumour choking the pelvis. These incarcerated tumours become moulded to the pelvis and present some operative difficulty, and hence the need for prompt relief in the early stage of a tumour confined to the pelvis in its development.

Owing to the presence of endometritis or a diseased condition of the ovaries and tubes, which so commonly accompany myo-fibromata, sterility is fortunately common. The coexistence of **pregnancy** with uterine myo-fibroma is a serious condition, but bearing in mind that two lives are involved, it appears unjustifiable to adopt radical measures unless the symptoms are urgent.

Before the child is viable operation may be required because of excessive pain or rapid growth, as in non-pregnant cases. It is worth remembering that in some of these cases the myo-fibroma may be shelled out without interfering with the normal course of pregnancy. So, too, when the tumour occupies such a position that delivery cannot be effected *per vias naturales* operation must be undertaken, though some pelvic tumours with a long pedicle can be displaced upwards into the abdomen, where they no longer menace the safe continuation of pregnancy and delivery.

If, however, the patient has gone almost to full term with a myo-fibroma which would, if left, require removal at a subsequent operation, it is better to effect delivery of the child by Cæsarean section, and then proceed to the removal of the myo-fibromatous uterus.

These, then, are the various considerations which should guide us in the selection of cases for operation. If we decide on operation, the best time is about a week before the expected "period," but in all cases it is desirable that the patient should be brought into the best possible condition before any operation is undertaken.

Myomectomy is the operation of choice in young women, provided that the general condition is fairly satisfactory, that the tumour does not extend much above the umbilicus, and that there are no important complications such as severe inflammatory disease of the tubes and ovaries or advanced anæmia.

Within proper limits this operation of shelling out the tumour should always be adopted, as by this means the functional activity of the uterus is unimpaired.

In cases where myomectomy is undesirable I prefer the operation of retro-peritoneal hystero-myomectomy rather than the so-called pan-hystero-myomectomy. When the myo-fibroma is associated with malignant disease nothing short of the removal of the whole uterus and tumour would be advisable, whereas in ordinary uncomplicated cases of hystero-myomectomy there is no *primâ facie* reason for the removal of any more of the uterus and tumour than is included in the supra-vaginal operation of retro-peritoneal hystero-myomectomy; but though we may regard the last-mentioned operation as the method of election, it would be foolish to insist on a slavish adherence to it under all circumstances.

These various procedures are of such severity, that they should not be undertaken if any grave organic mischief in other organs coexists. Advanced disease of the heart, lungs, or kidneys should certainly be regarded as contra-indications to operation.

